# Crystal structure of 1,10-phenanthrolinium 3-hy­droxy-2,4,6-tri­nitro­phenolate

**DOI:** 10.1107/S2056989015010737

**Published:** 2015-06-13

**Authors:** Selvarasu Muthulakshmi, Doraisamyraja Kalaivani

**Affiliations:** a PG and Research Department of Chemistry, Seethalakshmi Ramaswami College, Tiruchirappalli 620 002, Tamil Nadu, India

**Keywords:** crystal structure, spectroscopic characterization, sensitivity test, thermal testing, high energy density material, IHDEM

## Abstract

In the title molecular salt, C_12_H_9_N_2_^+^·C_6_H_2_N_3_O_8_^−^, the cation and anion are connected by an N—H⋯O hydrogen bond. In the anion, an intra­molecular O—H⋯O hydrogen bond with an *S*(6) ring motif is observed. The planes of two of the nitro groups are approximately parallel to the plane of the benzene ring, making dihedral angles of 3.9 (2) and 15.3 (2)°, while the third nitro group is almost perpendicular to the benzene ring, with a dihedral angle of 78.6 (3)°. In the crystal, cation–anion pairs related by an *n*-glide plane are connected by C—H⋯O hydrogen bonds, forming a chain structure along [101]. Sensitivity tests and thermal testing indicate that the title salt is an insensitive high-energy-density material (IHEDM).

## Abstract 2

The 1,10-phenanthrolinium cation and 3-hy­droxy-2,4,6-tri­nitro­phenolate anion are held together through an N—H⋯O hydrogen bond. In the crystal, cation–anion pairs are connected by C—H⋯O hydrogen bonds, forming a chain structure along [101]. Spectroscopic data also support the formation of a mol­ecular salt. Sensitivity tests and thermal testing indicate that it is an insensitive high energy density material (IHEDM).

## Chemical context   

2,4,6-Tri­nitro­benzene-1,3-diol (styphnic acid) is an energetic mol­ecule, which forms complexes with metal ions (Liu *et al.*, 2009 ▸; Zhang *et al.*, 2011 ▸; Zhu *et al.*, 2009 ▸) and salts with organic amines (Kalaivani & Malarvizhi, 2010 ▸; Kalaivani *et al.*, 2011 ▸; Muthulakshmi & Kalaivani, 2015 ▸; Srinivas *et al.*, 2014 ▸). 1,10-Phenanthroline is a well-known heterocyclic chelating agent (Goel & Singh, 2013 ▸; MacDonnell *et al.*, 1999 ▸). It also shows good anticancer activity (Sastri *et al.*, 2003 ▸). It is observed in the present study that although styphnic acid contains two acidic phenolic hydrogen atoms and 1,10-phenanthroline contains two basic tertiary nitro­gen atoms, they form only the monoprotonated title mol­ecular salt with 1:1 stoichiometry upon mixing of their ethano­lic solutions. 
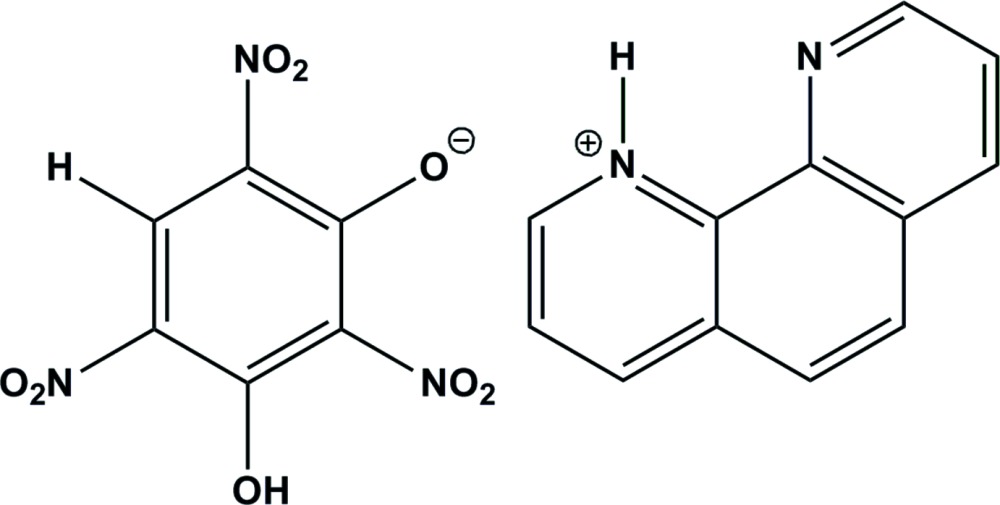


## Structural commentary   

**Figure 1:**
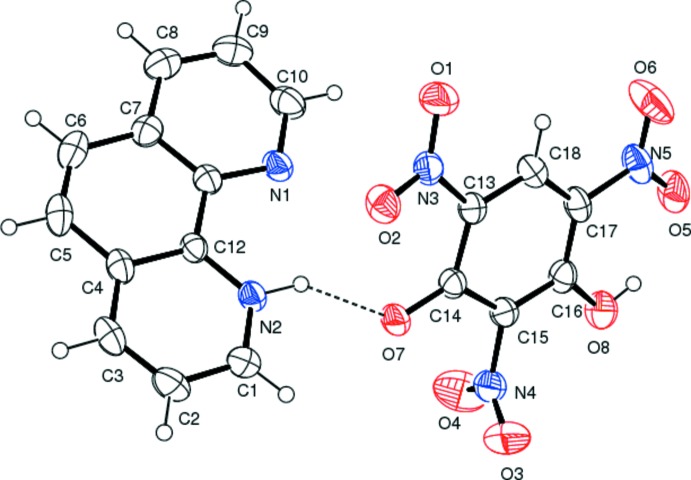
A view of the mol­ecular structure of the title mol­ecular salt, with the atom labelling. Displacement ellipsoids are drawn at the 40% probability level. The N—H⋯O hydrogen bond is shown as a dashed line.

The mol­ecular structure of the title mol­ecular salt is depicted in Fig. 1 ▸. The acidic hydrogen atom of the phenolic group in styphnic acid protonates the nitro­gen atom of 1,10-phenanthroline, making it a cation. An *S*(6) ring motif is formed in the anion by an intra­molecular O—H⋯O hydrogen bond (Table 1 ▸). Of the three nitro groups present in the anion, the plane of the one which is involved in the intra­molecular hydrogen bond deviates only slightly from the plane of benzene ring [dihedral angle 3.94 (8)°] to which it is attached. The nitro group flanked between the C—O^−^ group and the O—H group deviates to a greater extent [dihedral angle 78.62 (1)°] than the remaining nitro group which is oriented between the C—H and C—O^−^ groups [dihedral angle 15.27 (7)°].

## Supra­molecular features   

**Figure 2:**
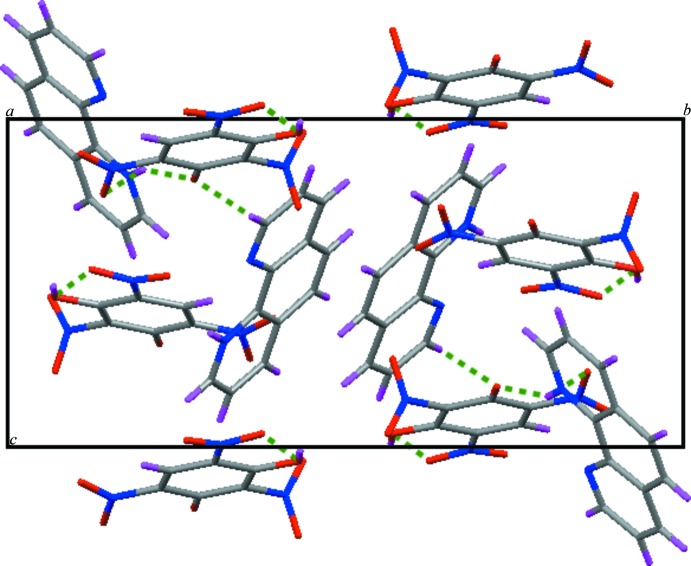
The crystal packing of the title mol­ecular salt viewed along the *a* axis. Hydrogen bonds are shown as dotted lines.

In the crystal, the C—O^−^ (acceptor) group of the phenolate anion and the N—H (donor) of the cation form an N—H⋯O hydrogen bond (Table 1 ▸ and Fig. 1 ▸). A weak C—H⋯O hydrogen bond is also observed in the crystal, forming a chain structure along [101] (Table 1 ▸ and Figs. 2 ▸ and 3 ▸).

## Database survey   

A search of the Cambridge Structural Database (Version 5.35, May 2014; Groom & Allen, 2014 ▸) for 3-hy­droxy-2,4,6-tri­nitro­phenolates gave 14 hits. Six concern metal-complex cations and eight organic cations. Amongst the latter are two compounds, referred to above in §1 for their high thermal stability, *viz*. 2-meth­oxy­anilinium 3-hy­droxy-2,4,6-tri­nitro­phenolate (Kalaivani *et al.*, 2011 ▸), morpholinium 3-hy­droxy-2,4,6-tri­nitro­phenolate (Kalaivani & Malarvizhi, 2010 ▸) while the crystal structure and thermal behaviour of pyridinium styphnate is reported by Muthulakshmi & Kalaivani (2015 ▸).

## Synthesis and crystallization   

Equimolar solutions of each of styphnic acid (2.45 g, 0.01 mol, 40 mL) and 1,10-phenanthroline monohydrate (1.98 g, 0.01 mol, 30 mL) in ethanol were mixed and shaken well for 3 h. On standing at 298 K for two h, the mixture yielded a yellow solid which was ground, washed well with dry ether and recrystallized from a ethanol–water mixture. Shining yellow single crystals were obtained from the mother liquor by slow evaporation (m.p. 395 K, yield 80%). Although the monoprotonated salt is obtained in good yield, several attempts to prepare the diprotonated salt from styphnic acid and 1,10-phenanthroline by mixing them in different concentrations in solvents of different polarity were not successful. The title mol­ecular salt is produced due to a proton-transfer reaction in which one of the two phenolic group hydrogen atoms is transferred to one of the tertiary nitro­gen atoms of 1,10-phenanthroline. This type of inter­action is also evidenced by the spectroscopic data [IR: 1532 (N—O asym. str.), 1297 (N—O sym. str.), 2200–3500, 461 (amine salt) cm^−1^ (Silverstein & Webster, 2004 ▸; Ramachandran *et al.* 2007 ▸); ^1^H NMR: δ 8.52 p.p.m. (*s*, C—H proton of phenolate moiety), 9.28–8.19 p.p.m. (*m*, ring proton of cation), 7.0–5.5 p.p.m. (broad, time-averaged signal of OH and NH protons); ^13^C NMR: δ 156.0, 148.1, 142.2, 138.0, 135.3, 129.9, 127.9, 126.1 and 126.0 p.p.m.].

## Sensitivity testing and thermal studies   

The title mol­ecular salt has three nitro groups attached to the benzene ring and hence it was subjected to sensitivity testing (impact sensitivity and friction sensitivity) and thermal studies (TGA/DTA). The mol­ecular salt is insensitive towards impact and friction (Meyer *et al.*, 2007 ▸). The activation energy for the decomposition of the title mol­ecular salt was determined from TGA/DTA curves obtained at four different heating rates (5, 10, 15 and 20 K min^−1^) applying Ozawa and Kissinger methods (Kissinger, 1957 ▸; Ozawa, 1965 ▸). The activation energy determined was 459 kJ mol^−1^ from the Ozawa plot and 478 kcal mol^−1^ from the Kissinger plot. The sensitivity tests and thermal studies indicate that this mol­ecular salt is an insensitive high-energy-density material (IHEDM).

## Refinement   

Crystal data, data collection and structure refinement details are summarized in Table 2 ▸. C- and O-bound H atoms were positioned geometrically with C—H = 0.93 Å and O—H = 0.82 Å, and were refined as riding with *U*_iso_(H) = 1.2*U*_eq_(C) and 1.5*U*_eq_(O). The N-bound H atom was located in a difference Fourier map and refined freely [N—H = 0.94 (2) Å].

## Supplementary Material

Crystal structure: contains datablock(s) global, I. DOI: 10.1107/S2056989015010737/is5402sup1.cif

Structure factors: contains datablock(s) I. DOI: 10.1107/S2056989015010737/is5402Isup2.hkl

Supporting information file. DOI: 10.1107/S2056989015010737/is5402Isup3.cml

CCDC reference: 1050845

Additional supporting information:  crystallographic information; 3D view; checkCIF report

## Figures and Tables

**Figure 3:**
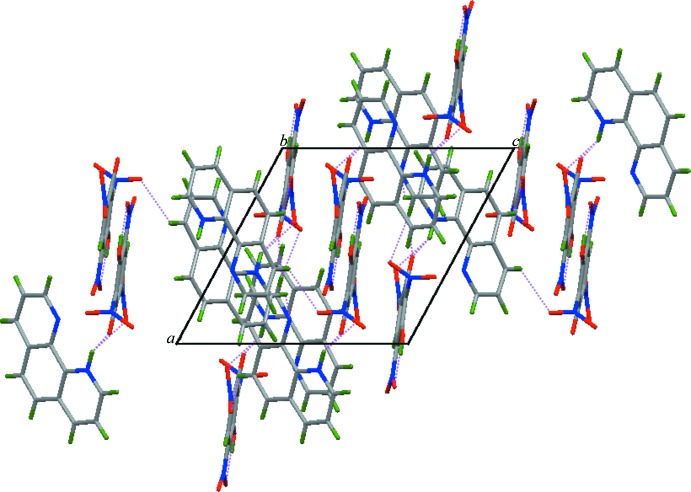
The crystal packing of the title mol­ecular salt viewed along the *b* axis. Hydrogen bonds are shown as dotted lines.

**Table 1:** Hydrogen-bond geometry (, )

*D*H*A*	*D*H	H*A*	*D* *A*	*D*H*A*
C10H10O7^i^	0.93	2.52	3.398(2)	158
N2H2*A*O7	0.94(2)	1.87(2)	2.702(2)	146.7(17)
O8H8*A*O5	0.82	1.88	2.579(2)	143

Symmetry code: (i) 
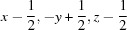
.

**Table 2:** Experimental details

Crystal data	
Chemical formula	C_12_H_9_N_2_^+^C_6_H_2_N_3_O_8_
*M*_r_	425.32
Crystal system, space group	Monoclinic, *P*2_1_/*n*
Temperature (K)	296
*a*, *b*, *c* ()	10.0984(7), 19.0072(14), 10.5124(7)
()	118.419(2)
*V* (^3^)	1774.6(2)
*Z*	4
Radiation type	Mo *K*
(mm^1^)	0.13
Crystal size (mm)	0.35 0.30 0.25

Data collection	
Diffractometer	Bruker Kappa APEXII CCD
Absorption correction	Multi-scan (*SADABS*; Bruker, 2004 ▸)
*T*_min_, *T*_max_	0.952, 0.970
No. of measured, independent and observed [*I* > 2(*I*)] reflections	35336, 4007, 2551
*R*_int_	0.040
(sin /)_max_ (^1^)	0.648

Refinement	
*R*[*F* ^2^ > 2(*F* ^2^)], *wR*(*F* ^2^), *S*	0.042, 0.125, 1.01
No. of reflections	4007
No. of parameters	284
H-atom treatment	H atoms treated by a mixture of independent and constrained refinement
_max_, _min_ (e ^3^)	0.24, 0.22

Computer programs: *APEX2* and *SAINT* (Bruker, 2004 ▸), *SIR92* (Altomare *et al.*, 1993 ▸), *SHELXL2014* (Sheldrick, 2015 ▸), *ORTEP-3 for Windows* (Farrugia, 2012 ▸) and *Mercury* (Macrae *et al.*, 2008 ▸).

## supplementary crystallographic information

### Crystal data

**Table:**

C_12_H_9_N_2_^+^·C_6_H_2_N_3_O_8_^−^	*F*(000) = 872
*M**_r_* = 425.32	*D*_x_ = 1.592 Mg m^−^^3^
Monoclinic, *P*2_1_/*n*	Mo *K*α radiation, λ = 0.71073 Å
*a* = 10.0984 (7) Å	Cell parameters from 8729 reflections
*b* = 19.0072 (14) Å	θ = 2.3–26.0°
*c* = 10.5124 (7) Å	µ = 0.13 mm^−^^1^
β = 118.419 (2)°	*T* = 296 K
*V* = 1774.6 (2) Å^3^	Plate, yellow
*Z* = 4	0.35 × 0.30 × 0.25 mm

### Data collection

**Table:**

Bruker Kappa APEXII CCD diffractometer	4007 independent reflections
Radiation source: fine-focus sealed tube	2551 reflections with *I* > 2σ(*I*)
Graphite monochromator	*R*_int_ = 0.040
ω and φ scan	θ_max_ = 27.4°, θ_min_ = 2.1°
Absorption correction: multi-scan (*SADABS*; Bruker, 2004)	*h* = −13→13
*T*_min_ = 0.952, *T*_max_ = 0.970	*k* = −24→24
35336 measured reflections	*l* = −13→13

### Refinement

**Table:**

Refinement on *F*^2^	0 restraints
Least-squares matrix: full	Hydrogen site location: mixed
*R*[*F*^2^ > 2σ(*F*^2^)] = 0.042	H atoms treated by a mixture of independent and constrained refinement
*wR*(*F*^2^) = 0.125	*w* = 1/[σ^2^(*F*_o_^2^) + (0.0575*P*)^2^ + 0.5078*P*] where *P* = (*F*_o_^2^ + 2*F*_c_^2^)/3
*S* = 1.01	(Δ/σ)_max_ < 0.001
4007 reflections	Δρ_max_ = 0.24 e Å^−^^3^
284 parameters	Δρ_min_ = −0.22 e Å^−^^3^

### Special details

**Table:**

Geometry. All esds (except the esd in the dihedral angle between two l.s. planes) are estimated using the full covariance matrix. The cell esds are taken into account individually in the estimation of esds in distances, angles and torsion angles; correlations between esds in cell parameters are only used when they are defined by crystal symmetry. An approximate (isotropic) treatment of cell esds is used for estimating esds involving l.s. planes.

### Fractional atomic coordinates and isotropic or equivalent isotropic displacement parameters (Å^2^)

**Table:**

	*x*	*y*	*z*	*U*_iso_*/*U*_eq_	
C1	0.7553 (2)	0.19947 (11)	0.2956 (2)	0.0454 (5)	
H1	0.7460	0.2332	0.3549	0.054*	
C2	0.8951 (2)	0.17090 (12)	0.3325 (2)	0.0528 (5)	
H2	0.9799	0.1855	0.4160	0.063*	
C3	0.9067 (2)	0.12136 (12)	0.2455 (2)	0.0494 (5)	
H3	1.0001	0.1016	0.2703	0.059*	
C4	0.78005 (19)	0.09958 (10)	0.1189 (2)	0.0394 (4)	
C5	0.7858 (2)	0.04786 (11)	0.0239 (2)	0.0518 (5)	
H5	0.8777	0.0280	0.0436	0.062*	
C6	0.6600 (3)	0.02750 (11)	−0.0938 (2)	0.0518 (5)	
H6	0.6664	−0.0065	−0.1544	0.062*	
C7	0.5170 (2)	0.05664 (10)	−0.1282 (2)	0.0412 (4)	
C8	0.3825 (2)	0.03423 (11)	−0.2468 (2)	0.0512 (5)	
H8	0.3837	−0.0012	−0.3071	0.061*	
C9	0.2506 (2)	0.06479 (12)	−0.2725 (2)	0.0548 (6)	
H9	0.1604	0.0501	−0.3499	0.066*	
C10	0.2520 (2)	0.11849 (11)	−0.1817 (2)	0.0518 (5)	
H10	0.1608	0.1394	−0.2021	0.062*	
C11	0.50613 (19)	0.10956 (9)	−0.04079 (19)	0.0350 (4)	
C12	0.64071 (18)	0.13032 (9)	0.08508 (18)	0.0331 (4)	
N1	0.37497 (16)	0.14143 (8)	−0.06844 (17)	0.0422 (4)	
N2	0.63483 (17)	0.17898 (8)	0.17614 (16)	0.0370 (4)	
H2A	0.542 (2)	0.1997 (11)	0.155 (2)	0.049 (6)*	
C13	0.20014 (19)	0.22681 (10)	0.12247 (19)	0.0372 (4)	
C14	0.30718 (19)	0.28269 (10)	0.14818 (18)	0.0368 (4)	
C15	0.23831 (19)	0.35056 (10)	0.11961 (19)	0.0391 (4)	
C16	0.0886 (2)	0.36441 (10)	0.06754 (19)	0.0408 (4)	
C17	−0.00782 (19)	0.30634 (11)	0.0441 (2)	0.0427 (5)	
C18	0.0499 (2)	0.23906 (11)	0.07130 (19)	0.0420 (4)	
H18	−0.0146	0.2012	0.0545	0.050*	
N3	0.24894 (18)	0.15442 (9)	0.15365 (17)	0.0425 (4)	
N4	0.33779 (19)	0.41089 (9)	0.1449 (2)	0.0517 (4)	
N5	−0.16493 (18)	0.31559 (11)	−0.00773 (18)	0.0539 (5)	
O1	0.15283 (17)	0.10808 (8)	0.10657 (19)	0.0679 (5)	
O2	0.38275 (15)	0.14144 (7)	0.22911 (15)	0.0511 (4)	
O3	0.4138 (2)	0.43000 (11)	0.2680 (2)	0.0912 (6)	
O4	0.3418 (2)	0.43695 (11)	0.0422 (2)	0.0951 (7)	
O5	−0.21917 (15)	0.37586 (9)	−0.04103 (16)	0.0630 (4)	
O6	−0.24439 (17)	0.26421 (11)	−0.0213 (2)	0.0863 (6)	
O7	0.44347 (13)	0.27585 (7)	0.18477 (15)	0.0483 (4)	
O8	0.04242 (15)	0.43111 (7)	0.04180 (16)	0.0577 (4)	
H8A	−0.0489	0.4327	0.0110	0.087*	

### Atomic displacement parameters (Å^2^)

**Table:**

	*U*^11^	*U*^22^	*U*^33^	*U*^12^	*U*^13^	*U*^23^
C1	0.0381 (10)	0.0461 (12)	0.0484 (11)	−0.0042 (8)	0.0178 (9)	−0.0046 (9)
C2	0.0317 (10)	0.0658 (14)	0.0502 (12)	−0.0055 (9)	0.0109 (9)	−0.0016 (11)
C3	0.0292 (9)	0.0600 (13)	0.0577 (12)	0.0085 (9)	0.0196 (9)	0.0102 (10)
C4	0.0335 (9)	0.0398 (10)	0.0486 (11)	0.0076 (8)	0.0224 (8)	0.0089 (9)
C5	0.0480 (12)	0.0521 (13)	0.0637 (13)	0.0191 (10)	0.0333 (11)	0.0081 (10)
C6	0.0645 (14)	0.0421 (12)	0.0583 (13)	0.0117 (10)	0.0369 (12)	−0.0003 (10)
C7	0.0482 (11)	0.0351 (10)	0.0435 (10)	0.0012 (8)	0.0245 (9)	0.0034 (8)
C8	0.0637 (14)	0.0454 (12)	0.0458 (11)	−0.0074 (10)	0.0270 (10)	−0.0058 (9)
C9	0.0484 (12)	0.0593 (14)	0.0453 (12)	−0.0129 (10)	0.0129 (10)	−0.0050 (10)
C10	0.0338 (10)	0.0539 (13)	0.0577 (13)	−0.0006 (9)	0.0136 (10)	0.0035 (10)
C11	0.0334 (9)	0.0328 (9)	0.0399 (10)	0.0014 (7)	0.0184 (8)	0.0053 (8)
C12	0.0310 (9)	0.0300 (9)	0.0404 (9)	0.0024 (7)	0.0186 (8)	0.0047 (8)
N1	0.0295 (8)	0.0429 (9)	0.0490 (9)	0.0024 (7)	0.0146 (7)	0.0011 (7)
N2	0.0287 (8)	0.0375 (9)	0.0438 (9)	0.0027 (7)	0.0164 (7)	0.0008 (7)
C13	0.0345 (9)	0.0380 (10)	0.0394 (10)	0.0043 (8)	0.0177 (8)	0.0016 (8)
C14	0.0305 (9)	0.0415 (10)	0.0358 (9)	0.0038 (8)	0.0137 (8)	−0.0018 (8)
C15	0.0324 (9)	0.0386 (10)	0.0428 (10)	0.0034 (8)	0.0150 (8)	−0.0016 (8)
C16	0.0371 (10)	0.0436 (11)	0.0384 (10)	0.0112 (8)	0.0153 (8)	0.0025 (8)
C17	0.0295 (9)	0.0572 (13)	0.0405 (10)	0.0098 (9)	0.0158 (8)	0.0063 (9)
C18	0.0344 (9)	0.0514 (12)	0.0410 (10)	−0.0001 (8)	0.0185 (8)	0.0033 (9)
N3	0.0415 (9)	0.0420 (9)	0.0492 (9)	0.0023 (7)	0.0257 (8)	0.0008 (7)
N4	0.0406 (10)	0.0412 (10)	0.0687 (12)	0.0036 (8)	0.0221 (9)	−0.0038 (9)
N5	0.0327 (9)	0.0761 (14)	0.0513 (10)	0.0101 (9)	0.0187 (8)	0.0090 (9)
O1	0.0523 (9)	0.0437 (9)	0.1065 (13)	−0.0066 (7)	0.0367 (9)	−0.0094 (8)
O2	0.0416 (8)	0.0498 (8)	0.0574 (8)	0.0109 (6)	0.0200 (7)	0.0091 (7)
O3	0.0767 (12)	0.0996 (15)	0.0885 (13)	−0.0341 (11)	0.0323 (11)	−0.0422 (11)
O4	0.0991 (15)	0.0829 (14)	0.0965 (14)	−0.0256 (11)	0.0410 (12)	0.0204 (11)
O5	0.0387 (8)	0.0775 (12)	0.0660 (10)	0.0230 (8)	0.0194 (7)	0.0057 (8)
O6	0.0357 (8)	0.0942 (14)	0.1205 (16)	0.0024 (9)	0.0304 (9)	0.0257 (12)
O7	0.0296 (7)	0.0430 (8)	0.0689 (9)	0.0042 (6)	0.0206 (6)	−0.0054 (7)
O8	0.0441 (8)	0.0482 (9)	0.0739 (10)	0.0189 (7)	0.0225 (7)	0.0038 (7)

### Geometric parameters (Å, º)

**Table:**

C1—N2	1.325 (2)	C11—C12	1.429 (2)
C1—C2	1.384 (3)	C12—N2	1.353 (2)
C1—H1	0.9300	N2—H2A	0.94 (2)
C2—C3	1.356 (3)	C13—C18	1.367 (2)
C2—H2	0.9300	C13—N3	1.446 (2)
C3—C4	1.399 (3)	C13—C14	1.446 (3)
C3—H3	0.9300	C14—O7	1.247 (2)
C4—C12	1.404 (2)	C14—C15	1.428 (3)
C4—C5	1.422 (3)	C15—C16	1.366 (2)
C5—C6	1.341 (3)	C15—N4	1.463 (3)
C5—H5	0.9300	C16—O8	1.333 (2)
C6—C7	1.424 (3)	C16—C17	1.414 (3)
C6—H6	0.9300	C17—C18	1.378 (3)
C7—C11	1.402 (3)	C17—N5	1.422 (2)
C7—C8	1.403 (3)	C18—H18	0.9300
C8—C9	1.357 (3)	N3—O2	1.2230 (19)
C8—H8	0.9300	N3—O1	1.227 (2)
C9—C10	1.393 (3)	N4—O3	1.204 (2)
C9—H9	0.9300	N4—O4	1.207 (3)
C10—N1	1.320 (2)	N5—O6	1.228 (2)
C10—H10	0.9300	N5—O5	1.246 (2)
C11—N1	1.356 (2)	O8—H8A	0.8200
			
N2—C1—C2	120.31 (19)	N2—C12—C11	120.09 (15)
N2—C1—H1	119.8	C4—C12—C11	121.09 (16)
C2—C1—H1	119.8	C10—N1—C11	116.72 (17)
C3—C2—C1	119.13 (18)	C1—N2—C12	122.82 (16)
C3—C2—H2	120.4	C1—N2—H2A	117.7 (12)
C1—C2—H2	120.4	C12—N2—H2A	119.4 (12)
C2—C3—C4	120.94 (18)	C18—C13—N3	116.50 (17)
C2—C3—H3	119.5	C18—C13—C14	122.64 (17)
C4—C3—H3	119.5	N3—C13—C14	120.85 (15)
C3—C4—C12	118.00 (18)	O7—C14—C15	120.97 (17)
C3—C4—C5	123.26 (17)	O7—C14—C13	126.74 (17)
C12—C4—C5	118.74 (17)	C15—C14—C13	112.22 (15)
C6—C5—C4	120.69 (18)	C16—C15—C14	126.41 (17)
C6—C5—H5	119.7	C16—C15—N4	117.07 (16)
C4—C5—H5	119.7	C14—C15—N4	116.50 (15)
C5—C6—C7	121.53 (19)	O8—C16—C15	118.55 (18)
C5—C6—H6	119.2	O8—C16—C17	124.17 (16)
C7—C6—H6	119.2	C15—C16—C17	117.28 (17)
C11—C7—C8	117.12 (18)	C18—C17—C16	120.00 (16)
C11—C7—C6	119.94 (18)	C18—C17—N5	118.63 (19)
C8—C7—C6	122.93 (19)	C16—C17—N5	121.37 (18)
C9—C8—C7	119.38 (19)	C13—C18—C17	121.38 (18)
C9—C8—H8	120.3	C13—C18—H18	119.3
C7—C8—H8	120.3	C17—C18—H18	119.3
C8—C9—C10	119.21 (19)	O2—N3—O1	122.31 (16)
C8—C9—H9	120.4	O2—N3—C13	119.42 (16)
C10—C9—H9	120.4	O1—N3—C13	118.22 (16)
N1—C10—C9	123.93 (19)	O3—N4—O4	124.2 (2)
N1—C10—H10	118.0	O3—N4—C15	117.6 (2)
C9—C10—H10	118.0	O4—N4—C15	118.16 (19)
N1—C11—C7	123.58 (17)	O6—N5—O5	121.52 (17)
N1—C11—C12	118.47 (16)	O6—N5—C17	119.63 (19)
C7—C11—C12	117.95 (16)	O5—N5—C17	118.84 (19)
N2—C12—C4	118.80 (16)	C16—O8—H8A	109.5
			
N2—C1—C2—C3	−0.5 (3)	N3—C13—C14—O7	6.9 (3)
C1—C2—C3—C4	0.7 (3)	C18—C13—C14—C15	2.4 (3)
C2—C3—C4—C12	−0.3 (3)	N3—C13—C14—C15	−176.13 (15)
C2—C3—C4—C5	−180.0 (2)	O7—C14—C15—C16	174.20 (18)
C3—C4—C5—C6	178.0 (2)	C13—C14—C15—C16	−2.9 (3)
C12—C4—C5—C6	−1.7 (3)	O7—C14—C15—N4	−3.9 (3)
C4—C5—C6—C7	0.1 (3)	C13—C14—C15—N4	178.93 (16)
C5—C6—C7—C11	2.2 (3)	C14—C15—C16—O8	−177.46 (17)
C5—C6—C7—C8	−177.0 (2)	N4—C15—C16—O8	0.7 (3)
C11—C7—C8—C9	1.0 (3)	C14—C15—C16—C17	2.4 (3)
C6—C7—C8—C9	−179.84 (19)	N4—C15—C16—C17	−179.48 (17)
C7—C8—C9—C10	0.9 (3)	O8—C16—C17—C18	178.77 (17)
C8—C9—C10—N1	−1.2 (3)	C15—C16—C17—C18	−1.1 (3)
C8—C7—C11—N1	−2.8 (3)	O8—C16—C17—N5	−1.2 (3)
C6—C7—C11—N1	178.01 (17)	C15—C16—C17—N5	178.99 (17)
C8—C7—C11—C12	176.54 (17)	N3—C13—C18—C17	177.12 (16)
C6—C7—C11—C12	−2.7 (3)	C14—C13—C18—C17	−1.4 (3)
C3—C4—C12—N2	−0.4 (3)	C16—C17—C18—C13	0.7 (3)
C5—C4—C12—N2	179.32 (17)	N5—C17—C18—C13	−179.38 (17)
C3—C4—C12—C11	−178.65 (17)	C18—C13—N3—O2	−163.44 (16)
C5—C4—C12—C11	1.1 (3)	C14—C13—N3—O2	15.1 (3)
N1—C11—C12—N2	2.2 (2)	C18—C13—N3—O1	14.3 (2)
C7—C11—C12—N2	−177.13 (16)	C14—C13—N3—O1	−167.14 (17)
N1—C11—C12—C4	−179.56 (16)	C16—C15—N4—O3	103.1 (2)
C7—C11—C12—C4	1.1 (2)	C14—C15—N4—O3	−78.6 (2)
C9—C10—N1—C11	−0.5 (3)	C16—C15—N4—O4	−79.0 (2)
C7—C11—N1—C10	2.5 (3)	C14—C15—N4—O4	99.3 (2)
C12—C11—N1—C10	−176.79 (16)	C18—C17—N5—O6	3.0 (3)
C2—C1—N2—C12	−0.2 (3)	C16—C17—N5—O6	−177.05 (19)
C4—C12—N2—C1	0.6 (3)	C18—C17—N5—O5	−175.60 (17)
C11—C12—N2—C1	178.89 (17)	C16—C17—N5—O5	4.3 (3)
C18—C13—C14—O7	−174.58 (18)		

### Hydrogen-bond geometry (Å, º)

**Table:**

*D*—H···*A*	*D*—H	H···*A*	*D*···*A*	*D*—H···*A*
C10—H10···O7^i^	0.93	2.52	3.398 (2)	158
N2—H2*A*···O7	0.94 (2)	1.87 (2)	2.702 (2)	146.7 (17)
O8—H8*A*···O5	0.82	1.88	2.579 (2)	143

Symmetry code: (i) *x*−1/2, −*y*+1/2, *z*−1/2.
